# Manuscript title: Facilitators and barriers to cotrimoxazole prophylaxis among HIV exposed babies: a qualitative study from Harare, Zimbabwe

**DOI:** 10.1186/s12889-015-2136-0

**Published:** 2015-08-15

**Authors:** Euphemia L. Sibanda, Sarah Bernays, Ian V. D. Weller, James G. Hakim, Frances M. Cowan

**Affiliations:** Centre for Sexual Health and HIV/AIDS Research, (CeSHHAR) Zimbabwe, 9 Monmouth Rd, Avondale West, Harare, Zimbabwe; Department of Health Services Research and Policy, London School of Hygiene and Tropical Medicine, London, UK; Department of Infection and Population Health, University College London Medical School, London, UK; Department of Medicine, University of Zimbabwe College of Health Sciences, Harare, Zimbabwe

## Abstract

**Background:**

Implementation of cotrimoxazole prophylaxis (CTX-p) among HIV-exposed infants (HEI) is poor in southern Africa. We conducted a study to investigate barriers to delivery of CTX-p to HEI in Zimbabwe at each step of the care cascade. Here we report findings of the qualitative component designed to investigate issues related to adherence conducted among women identified as HIV positive whose babies were started on CTX-p postnatally. Of note, Zimbabwe also provided nevirapine prophylaxis for HIV exposed babies, so the majority were giving nevirapine and CTX-p to their babies.

**Methods:**

Between Feb–Dec 2011, the first 20 HIV infected mothers identified were invited for in-depth interview 4–5months postnatally. Interviews were recorded, transcribed, translated and analysed thematically.

**Results:**

All women desired their baby’s health above all else, and were determined to do all they could to ensure their wellbeing. They did not report problems remembering to give drugs. The baby’s apparent good health was a huge motivator for continued adherence. However, most women reported that their husbands were less engaged in HIV care, refusing to be HIV tested and in some cases stealing drugs prescribed for their wives for themselves. In two instances the man stopped the woman from giving CTX-p to the baby either because of fear of side effects or not appreciating its importance. Stigma continues to be an important issue. Mothers reported being reluctant to disclose their HIV status to other people so found it difficult to collect prescription refills from the HIV clinic for fear of being seen by friends/relatives. Some women reported that it was hard to administer the drugs if there were people around at home. Other challenges faced were stock-outs of CTX-p at the clinic, which occurred three times in 2011. The baby would then go without CTX-p if the woman could not afford buying at a private pharmacy.

**Conclusions:**

The study highlights that adherence knowledge and desire alone is insufficient to overcome the familial and structural barriers to maintaining CTX-p. Improving adherence to CTX-p among HEI will require interventions to improve male involvement, reduce HIV stigma in communities and ensure adequate supply of drugs.

## Background

Infants born to HIV infected mothers (HIV-exposed infants, HEI) are at risk of mother-to-child transmission of HIV during pregnancy, delivery and breastfeeding. There is also evidence that HEI suffer more morbidity and mortality than infants born to HIV negative mothers likely due to 1) greater exposure to infection agents, 2) poor nutrition and care as a result of the mother’s illness or death [1–3]; and 3) altered innate immunity which could be mediated by exposure to HIV in utero and early in life or exposure to maternal antiretroviral drugs during prevention of mother to child transmission (PMTCT) [4, 5]. Many interventions have been introduced to prevent mother-to-child transmission of HIV and/or opportunistic infections among both HIV-infected and uninfected HEI. These include ARV prophylaxis and/or treatment regimens for the mother and infant that are given for various lengths of time depending on the infant feeding method and the WHO guideline that the PMTCT programme has adopted. Those programmes adopting Option B/B+ guidelines advise HEI to take ARV prophylaxis for the first 4–6 weeks of life, but those adopting Option A recommend that infants continue on nevirapine prophylaxis until one week after cessation of breastfeeding [6, 7]. In addition, all HEI in generalised HIV epidemics should take cotrimoxazole prophylaxis (CTX-p) from the first 4–6 weeks of life and continue until HIV infection can be excluded [8]. This means mothers and carers need to devote attention and care in ensuring adherence to these life-saving pharmacological interventions.

Unfortunately, current literature shows an unacceptable loss to follow up of infants along the PMTCT cascade [9, 10]. A recent systematic review estimated that 34 % of infants in Sub-Saharan Africa are lost to follow up from real-life PMTCT programs by three months of age, and 45 % are lost after HIV testing [11]. In Zimbabwe, retention of HEI has been a challenge since inception of PMTCT services. In 2007, only 11 % of infants who needed CTX-p received it [12] increasing to 34 % in 2010 [13] and 55 % in 2012 [14]. Studies have been done to explore barriers to retention of HEI along the PMTCT cascade from the carer’s perspective [15–17], and these include intrapersonal barriers such as fear that the infant will test HIV positive, spiritual and traditional beliefs, disbelief that PMTCT interventions work, time and money constraints and fear of disclosure of HIV status; interpersonal and community e.g. unsupportive male partners, stigmatising attitudes from family and community; health care system e.g. unfriendly health care workers, long queues and waiting times and different appointments for mothers and infants. However, there is a dearth of literature on barriers and facilitators to adherence to pharmaceutical interventions among HEI who are retained in regular follow-up.

As part of a wider study that investigated barriers at various steps of the PMTCT cascade, we conducted a qualitative study to explore women’s ability to maintain their babies on CTX-p after they had been initiated at age six weeks. Although this study focused on CTX-p, the findings and implications do not relate to the issue of CTX-p provision alone but to the whole package of services provided to HIV-exposed infants along the PMTCT cascade.

## Methods

The study was conducted at Mbare Clinic, Harare, during the period when the country was implementing WHO Option A (Zimbabwe transitioned to option B+ between 2013 and 2014). Between February to December 2011, we consecutively enrolled twenty willing women (who gave informed consent) and individually interviewed them in-depth. Eligible women were HIV positive and had babies who been started on CTX-p at age six weeks. In addition to CTX-p most babies were also being prescribed nevirapine prophylaxis (babies whose mothers were on ARVs for their own health were not prescribed nevirapine prophylaxis after six weeks of age). To identify HIV positive women, a cross-sectional survey was conducted during the antenatal period. A female study nurse conducted the survey and a female pharmacist who had been trained in social science methods conducted the in-depth interviews. Both study staff were external to the clinic system (i.e. they were not employed at the clinic and participants understood this).

Interviews were held in private at the clinic when the baby was 4–5 months old. Women were interviewed in their local language according to an interview guide which explored perception of their HIV status, views on importance of CTX-p, and barriers and facilitators to medication adherence. Table 1 shows the key areas of investigation. Given the sensitive nature of discussions on HIV, in-depth interviews were the method of choice because they allow exploration of participant experiences or social contexts in private. Participants were assured that researchers would handle all data with confidentiality; interview records were identified using codes/numbers instead of names.

**Table 1:** Key areas of investigation during in-depth interviews

Key areas of investigation	Specific probes
Background information	Area of residence, marital status, family environment
Current health status	Physical strength and well-being following delivery
Feelings about HIV infection	History of HIV testing; feelings about HIV positive diagnosis
HIV care for the participant (mother)	What HIV care has been sought
Views of risk of mother-to-child transmission of HIV	Awareness/knowledge of risks; feelings about ability to protect the baby from infection
Views on cotrimoxazole prophylaxis	Knowledge/awareness of the importance of cotrimoxazole prophylaxis; views on CTX-p
Experience giving CTX-p to the baby	Barriers and facilitators to daily administration, including experience with prescription refills at the clinic
Awareness of other PMTCT services that baby is supposed to get	Probes on awareness
Any related issues that participant wanted to raise	

Interviews were completed in 30—45 min. All were digitally recorded, transcribed, translated and analysed according to thematic analysis. Data analysis commenced as soon as data collection began: after each interview field notes were written with a view to identifying emerging themes. Following transcription and translation, analytic summaries were written for each interview using an approach which compared themes across participants, thus identifying similarities and deviant cases. A coding framework was developed and used for coding all the interviews. Coding was done by one researcher. Data handling and analysis was done using NVIVO 10 (qualitative data management software). In writing the report, thematic findings were illustrated using verbatim quotes which were selected on the basis of being representative of the quotes that related to a specific theme.

### Ethical considerations

The study had ethical approval from Medical Research Council of Zimbabwe and University College London Research Ethics Committee. Written informed consent was obtained from all participants before interviews were conducted.

## Results

### Description of participants

Out of 35 HIV positive women who were identified during the cross-sectional survey, 20 agreed to regular follow-up (and were interviewed at 4–5 months for this qualitative study); four were lost to follow-up before the 4–5 month period and 11 refused participation. Most refusers said they had no time. Non-participants did not differ from participants in terms of demographic characteristics.

To give an idea of the characteristics of the 20 interviewed participants: most (16) were married and majority were multiparous (Table 2). Thirteen were diagnosed with HIV during their most recent pregnancy, four in previous pregnancies and three had been diagnosed as a result of theirs or their children’s illness. All came from poor families; they either had no own income or earned very little from self-employment. The women lived in crowded accommodation where it was common to find several people (or more than one family) residing in a single room.

**Table 2:** Characteristics of interviewed participants

Participant	Age (Years)	Marital Status	No of children	Timing of HIV diagnosis	Occupation
1	30	Married	4	During ANC^a^, at 3 months	Sells vegetables
2	23	Married	2	During ANC	Not employed
3	35	Married	3	During ANC, at 6 months	Sells vegetables
4	36	Married	2	Previous pregnancy, 2009	Not employed
5	33	Married	3	During ANC, at 9 months	Sells vegetables
6	33	Married	2	Previous pregnancy, 2003	Not employed
7	37	Married	4	During ANC, at 6 months	Sells vegetables
8	27	Married	3	During ANC, at 8 months	Not employed
9	41	Divorced	5	2005 when she was sick	Meat vending
10	19	Single	1	During ANC, at 6 months	Not employed
11	35	Married	3	During ANC, at 7 months	Not employed
12	23	Married	1	Previous pregnancy, 2009	Not employed
13	24	Married	2	VCT^b^, March 2010	Not employed
14	24	Married	3	During ANC, at 8 months	Meat vending
15	29	Divorced	3	Tested at delivery	Dancer
16	33	Widow	5	During ANC, at 8 months	General vending
17	27	Married	4	2008, child was sick	Sells vegetables
18	23	Married	1	During ANC, at 6 months	General vending
19	32	Married	2	2007, when she was sick	Sex work
20	35	Married	4	Previous pregnancy, 2008	Sells vegetables

^a^ANC, Antenatal care

^b^VCT, Voluntary Counselling and Testing

### Barriers and facilitators to CTX-p adherence

Factors affecting women’s ability to keep their babies on CTX-p were grouped into 1) participant-level, 2) personal and community relationship level, and 3) health care system factors.

### Participant knowledge, attitudes and beliefs

#### Desire for baby’s health

In all interviews, it was clear that women were anxious about how their HIV infection might affect their babies’ health; they reported being determined to do their best to ensure the baby’s wellbeing. They understood the importance of CTX-p for the baby’s health and reported that they never forgot to administer the drug.

*It causes you to constantly be alert; this is a baby I love, it’s a life…since the nurses who have the experience said it works so I have to make it possible.****24-year old married mother of two***

#### Perceived health benefit

The perceived benefits of CTX-p encouraged women to keep giving it. During interviews they marvelled at how much healthier their babies were compared to other children in the community or their older children who had not been on CTX-p.

*I have noticed that giving cotrimoxazole to the baby is helpful. If you keep giving it he does not suffer from colds, or diarrhoea like what other children do.****27-year old married mother of four***

*Cotrimoxazole is important because in the community I live there are some childrenwho are not on medication, as I speak right now they have been to hospital a countless number of times. But with this child I have not been to hospital since delivery.****35 year old, married mother of four***

Having a baby test HIV negative at six weeks was reported to provide encouragement and further impetus to keep giving cotrimoxazole and nevirapine, likely because of increased confidence in the health care interventions that were offered at the clinic.

*When I was given her HIV (negative) test results that’s when I became more convinced that it is important to adhere to the given instructions.****24 year old married mother of two***

Unfortunately, the converse was also true; perceived ineffectiveness of PMTCT interventions was associated with poor adherence. One woman stopped giving CTX-p when her baby tested HIV positive because she felt let down by the pharmaceutical interventions.

#### Aids to remembering

Because adherence was important to them, women reported that they used reminders to ensure that they did not forget. Most used phone alarms as aids to remembering. As many had already established routines for taking their own medicines (ARVs and CTX-p) they found it easy to fit the babies into these routines.

#### Acceptance of HIV results

All participants reported that they were devastated when they themselves tested HIV positive; two said they had contemplated suicide. Most had initially interpreted the diagnosis as warning of imminent death and some had not known that it was possible to give birth to an HIV negative baby.

*…I thought, is this really my status? Does this really mean I am about to die? All my thoughts were about death.****24 year old married mother of two***

Fortunately, most participants reported that their initial fears were replaced by more positive thoughts following support, counselling and education from family and health care workers, as described below. Three women did not get this support and had not come to terms with their HIV status by the time of the interview. There was evidence that those women who had not come to terms with their status reported adherence challenges, e.g. not collecting prescription refills on time and not giving medicine when they were feeling depressed.

*I am putting effort to block it out of my mind. I heard some people say if you think about it too much, he-e-e your mind…you will get stressed and you will die quickly. So I pretend it doesn’t matter and ignore it…****29-year old divorced mother of three, sometimes didn’t collect prescription refills***

### Personal and community relationships

Personal and community relationships had a direct impact on adherence and were also important for acceptance of HIV status (which itself was associated with good adherence).

#### Relations with husbands/partners

Analysis of women’s accounts revealed that husbands/partners (16 participants were married) had a make-or-break influence on adherence. When looking at patterns in the women’s responses on level of adherence support they received from their husbands, it was clear that the less engaged in HIV issues a husband was, the less supportive for CTX-p. Three groups of husbands/partners were identified: 1) six men who provided the most support for adherence, 2) five who were not opposed to CTX-p but did not actively support it, and 3) five who opposed CTX-p.

Out of the 16 married women, only six had husbands who were positively supportive of CTX-p. The main factor that distinguished these men was that they had been for couples testing and counselling together with their wives. Two were HIV negative, and the others were either already on ART or in regular monitoring for ART eligibility. These men supported adherence in many ways. First, they provided support to their wives during the difficult period soon after HIV diagnosis which helped the women accept their HIV status which was apparently important for adherence.

*With time I accepted it (*her HIV status) *because when I told him he received it well and we went for couples testing and he accepted it.****23 year old mother of one***

*I was expecting that he would start shouting at me, saying how come you have the disease, where did you get it from? But he was the one to say ahh it is possible to get a situation where the man has no disease and the woman has it…So because he did not get angry the situation did not become too difficult for me; because my husband showed me love, and said let’s remain together.****Married mother of four, HIV sero-discordant relationship***

This group of men also took an active part in ensuring that the baby was given CTX-p, e.g. by reminding the woman to give it and buying it from private pharmacies when the clinic had no supplies.

The second group consists of five men who had taken small steps towards confronting HIV in the family. All initially had negative reactions when the women disclosed their HIV results but with time they came to a point where they could discuss their family’s HIV care; they accepted the wife’s HIV status and were not opposed to the baby taking CTX-p, although they did not actively support it. Their main challenge was that they were not ready to share their own HIV status with their wives. All of them refused to go for couples counselling and testing and were sometimes hostile when this was suggested. There was the suggestion that the response to HIV was shaped by relationship dynamics and gender norms; it seemed that men were more accepting of their partner’s HIV status than the women expected. When explaining why her husband did not want to test for HIV, one participant said *“He is afraid that if he tests positive I might leave him,”* which is a paradox given that he had not left her when he discovered that she had HIV.

The least supportive group consisted of five husbands who were opposed to CTX-p. They reportedly did not want any reference to HIV in the family. When their wives told them their results they refused to accept them and refused to understand the reasons why the baby had to be on cotrimoxazole; one feared that cotrimoxazole had harmful side effects. Three of the husbands forced their wives to stop giving CTX-p.

*I explained that the cotrimoxazole is for prevention of infections. And he said ha-a my child is not going to keep taking medicines willy-nilly.****23-year old married mother of two***

One participant tried to keep giving the CTX-p in secret but when her husband discovered it *“He said if I kept giving cotrimoxazole he would take the baby away from me and give him to his mother*.”

Thirteen women were in regular HIV care for their own health, and were taking CTX-p. Two of the seven women who were not in regular HIV care reported that they were stopped from taking their cotrimoxazole by their husbands, while ironically two men reportedly stole the CTX-p that had been prescribed for their wives for themselves.

*About a month after I delivered he suddenly asked me what the tablets (cotrimoxazole) were for. I explained to him and he became angry and said, “This is madness, these are all lies.” Then he took the tablets and threw them away.”****23-year old married mother of two***

#### Relationships in the community: fear of HIV disclosure

The community was reported as not having good understanding of HIV, and did not have a positive attitude towards HIV positive people, who were often laughed at or ridiculed. Women were therefore keen to prevent unwanted HIV disclosure, sometimes even to people who lived in the same crowded room, in order to avoid stigma. This made it difficult to give medicine at home.

“*The challenge is people can come before I have given the medicine; then I will take the bottle outside… I can actually go and give the medicine in the toilet”.****23-year old married mother of one***

This was particularly a challenge because of the unhygienic state of the shared toilets.

The same fear of stigma also made it difficult to collect prescription refills at the clinic. Although all chronic disease patients collected their medicines from this clinic, the community viewed it as an HIV clinic which made it difficult for some women to go there.

*Sometimes it’s hard for me to collect the medicine because I might see someone I know; then it becomes incredibly difficult because I have not disclosed to anyone except my husband.****23-year old married mother of one***

### Health system factors

#### Counselling and education

The education and counselling that health care workers gave soon after HIV diagnosis was reported to be helpful in allaying fears and helping women accept their HIV status.

*She (the counsellor) helped me to accept it…she told me that I can give birth to a normal baby who can grow up without HIV.****23 year old mother of one***

Of note, none of the women reported that they got this support/education from health care workers at the clinic, but they had either visited other centres or been counselled/educated by the study nurse.

Health system barriers to adherence included drug stock-outs and long queues at the clinic.

#### Drug stock-outs

The wider study in which this qualitative work was nested found that paediatric cotrimoxazole went out of stock three times in the course of 2011; in two instances it was out for almost three weeks. Nine of the twenty women reported having been affected by the stock-outs, which meant they had to buy from private pharmacies. Babies whose mothers could not afford buying went without medicine. Related to this, several participants reported that they sometimes had insufficient cotrimoxazole prescribed to last until their next appointment; many said they would get 100mls (40 days’ supply) yet the date of the next appointment was two months away, thereby forcing poor adherence practices.

*I stopped giving cotrimoxazole at that time. I could not give it as directed because I wanted it to last until the next appointment date.****41 year old divorced mother of five***

### Long queues at the clinic

Long queues/waiting times at the clinic also posed challenges to women. Participants reported that they typically had to dedicate the whole day to a clinic visit, which they found difficult.

*We just know that a clinic appointment means that you will spend the whole day at the clinic; you will not even have time to wash the baby’s nappies.****41 year old married mother of 5***

Related to this, women were scheduled to attend the clinic at different times for their own HIV care, which meant spending more days at the clinic.

## Discussion

The study revealed that adherence to CTX-p among HEI is influenced by the mother’s knowledge, attitudes and beliefs including perception of the importance and effectiveness of the intervention, desire for the baby’s health and acceptance of HIV status. Husbands were reported to have an important role, and the fear of HIV disclosure and subsequent stigma limited women’s ability to freely give CTX-p at home or to collect prescription refills. Health system factors such as provision of education and counselling and availability of drugs were important determinants of adherence. A significant strength of this study was the trust that participants had in the researcher/interviewer, whom they understood to be a health care worker who had a lot of information on HIV. As a result they openly discussed intimate details about their lives in the way they would with a trusted health care worker, sometimes in an effort to get more assistance/guidance on what services to seek for themselves or their babies and sometimes as to vent their feelings (in some instances the study team were the first people that the women had an in-depth discussion about their HIV infection with). The fact the interviewer was known to be external to the system at the clinic helped participants feel able to openly discuss challenges they faced with the clinic. This allowed a clearer insight into the participant’s situations. Although this study focused on CTX-p, the findings can be generalised to other pharmaceutical PMTCT interventions.

As expected, awareness of the rationale for giving CTX-p was associated with good adherence, which underlies the importance of educating mothers and carers about CTX-p/PMTCT interventions that have been prescribed. Perception of effectiveness is an important factor that motivated mothers: where the intervention was perceived to be ineffective, poor adherence ensued as was seen when one woman lost faith in all pharmaceutical interventions after her baby tested HIV positive. Clearly she had not properly understood the different roles of nevirapine and CTX-p and did not adequately appreciate the concept of risk reduction (as compared to risk cessation). It is likely important that women are given a more nuanced understanding of the likely effectiveness of nevirapine and CTX-p to ensure ongoing compliance in the event of transmission events. In the event of transmission, health care workers need to revisit this and also counsel about the importance of ongoing prophylactic treatment.

The extent of male partner’s engagement in HIV issues more generally was found to be a strong determinant of male support for CTX-p adherence. These findings add to the literature around importance of male involvement for PMTCT and provide an explanation of previous research findings that showed an increase in uptake of PMTCT services with couples testing and counselling [18–21]. Couples counselling improves the couple’s joint understanding of HIV and facilitates acceptance of HIV status and the family’s engagement in HIV treatment and prevention. Importantly, there was suggestion that men who had not received education/counselling thought HIV was emasculating, a finding that has been reported in other studies in Zimbabwe [22, 23]. It is clearly important to find ways to make couples testing and counselling acceptable to both parties. Education/messaging that emphasises how HIV testing can help a man achieve his ‘manly’ roles may be beneficial, e.g. accessing treatment after testing HIV positive will help maintain the man’s health and enable continued participation in masculine roles like providing for the family.

The fear of stigma was an important factor that limited women’s ability to give medicine at home or to collect prescription refills from the clinic. Of importance is the reminder that stigma also exists within marital relationships, hence the importance of stigma-reduction interventions cannot be overestimated. In addition to interventions to reduce stigma more generally, it may be worth considering if any small changes at the clinic could reduce the stigma associated with pharmacy refill- for example integration of services as described below. Stigma interventions have been classified into four categories: 1) informational approaches to give information on HIV disease, transmission and methods of reducing risk of acquisition, e.g. through mass media, educational lectures etc; 2) skill building, where potential perpetrators of stigma are given skills of how to interact with HIV positive people, 3) counselling/support to help HIV positive individuals to cope with the disease, e.g. in support groups, and 4) testimonials from people living with HIV [24, 25]. Many stigma-reduction activities are already going on in Zimbabwe, e.g. mass media campaigns, teaching about HIV in schools, and through the National Behaviour Change Programme, but more still needs to be done to achieve communities with low stigma levels. Stigma reduction is particularly important for PMTCT programs: mathematical modelling estimates that failure to access PMTCT services as a result of stigma may account for more than half (53 %) of all perinatal HIV transmissions, and that scale-up of stigma reduction interventions has the potential to avert 33 % of vertical infections [26, 27].

The health care system was seen to play an important role in adherence—stock-outs of cotrimoxazole resulted in many missed CTX-p doses. Findings in the larger study indicated that there was more than enough stock of cotrimoxazole nationally, but that logistic and training challenges interrupted the supply chain. Training local clinic staff on supply chain management will alleviate these challenges. The long waiting times at the clinic were also reported to be problematic, particularly since the mothers had to wait separately for their own HIV care. Integration of mother/baby HIV care might help reduce this burden on mothers. Still on the subject of integration, although the clinic has taken steps to prevent HIV disclosure by integrating HIV services within the general chronic diseases clinic, many people still perceive the integrated unit as an HIV clinic, which limits women’s ability to freely collect prescription refills for their babies. Integration of all services into the general outpatient clinic might be one way of solving this challenge.

### Limitations

The qualitative study was conducted in a small group of women in Mbare. Although the findings were insightful and consistent across participant accounts, they cannot be generalised to the whole Zimbabwean population. Information on husband/partner attitudes towards CTX-p adherence was obtained from the interviewed women, not the husbands themselves.

## Conclusions

The study highlights that adherence knowledge and desire to protect one’s child from HIV is insufficient to overcome the familial and structural barriers to maintaining CTX-p. Improving adherence among HEI will require interventions to improve male engagement with HIV issues and to increase acceptance of couples counselling and testing, reduce HIV stigma in communities and ensure continuous supply of drugs.
